# Paediatric non-progression following grandmother-to-child HIV transmission

**DOI:** 10.1186/s12977-016-0300-y

**Published:** 2016-09-08

**Authors:** M.-H. Tsai, M. Muenchhoff, E. Adland, A. Carlqvist, J. Roider, D. K. Cole, A. K. Sewell, J. Carlson, T. Ndung’u, P. J. R. Goulder

**Affiliations:** 1 Department of Paediatrics, University of Oxford, Peter Medawar Building for Pathogen Research, South Parks Road, Oxford, OX1 3SY UK; 2 Cardiff University School of Medicine, Heath Park, Cardiff, UK; 3 Microsoft Research, eScience Group, Los Angeles, CA USA; 4 HIV Pathogenesis Program, Doris Duke Medical Research Institute, University of KwaZulu-Natal, Durban, South Africa; 5 Ragon Institute of Massachusetts General Hospital, Massachusetts Institute of Technology and Harvard University, Cambridge, MA USA; 6 Max Planck Institute for Infection Biology, Berlin, Germany

**Keywords:** HIV, HLA-B*81:01, Paediatric non-progressor, Grandmother-to-child transmission, Viral replicative capacity, CT

## Abstract

**Background:**

In contrast to adult HIV infection, where slow disease progression is strongly linked to immune control of HIV mediated by protective HLA class I molecules such as HLA-B*81:01, the mechanisms by which a minority of HIV-infected children maintain normal-for-age CD4 counts and remain clinically healthy appear to be HLA class I-independent and are largely unknown. To better understand these mechanisms, we here studied a HIV-infected South African female, who remained a non-progressor throughout childhood.

**Results:**

Phylogenetic analysis of viral sequences in the HIV-infected family members, together with the history of grand-maternal breast-feeding, indicated that, unusually, the non-progressor child had been infected via grandmother-to-child transmission. Although HLA-B*81:01 was expressed by both grandmother and grand-daughter, autologous virus in each subject encoded an escape mutation L188F within the immunodominant HLA-B*81:01-restricted Gag-specific epitope TL9 (TPQDLNTML, Gag 180–188). Since the transmitted virus can influence paediatric and adult HIV disease progression, we investigated the impact of the L188F mutant on replicative capacity. When this variant was introduced into three distinct HIV clones in vitro, viral replicative capacity was abrogated altogether. However, a virus constructed using the *gag* sequence of the non-progressor child replicated as efficiently as wildtype virus.

**Conclusion:**

These findings suggest alternative sequences of events: the transmission of the uncompensated low fitness L188F to both children, potentially contributing to slow progression in both, consistent with previous studies indicating that disease progression in children can be influenced by the replicative capacity of the transmitted virus; or the transmission of fully compensated virus, and slow progression here principally the result of HLA-independent host-specific factors, yet to be defined.

**Electronic supplementary material:**

The online version of this article (doi:10.1186/s12977-016-0300-y) contains supplementary material, which is available to authorized users.

## Background

In the absence of antiretroviral therapy (ART), HIV infected children normally progress to disease rapidly, approximately 50 % developing AIDS by 1 year of life, and by 2 years of life 50 % have died. In adult infection these times are 10 and 11 years, respectively [1]. For these reasons, in 2013 the WHO have recommended that ART should be initiated in all HIV-infected children aged <5 years, and also in HIV-infected children whose absolute CD4 counts are <500 cells/mm^3^ (http://www.who.int/hiv/pub/guidelines/arv2013/intro/executivesummary/en). There remain, however, a number of ART-naïve, HIV-infected children, aged <5 years whose absolute CD4 counts are >500 cells/mm^3^ and who represent approximately 10–15 % of infected children, who are relative slow progressors [2–4]. Approximately one-third of the children within this group, therefore ~5 % of all infected children, have absolute CD4 counts of >750 cells/mm^3^, that are therefore indistinguishable from those of age-matched HIV-uninfected children (10th–90th centiles; [5, 6]). Study of these slow-progressor children provides the opportunity to gain insights into mechanisms of non-pathogenicity in HIV infection that appear to differ substantially from those operating in adult infection [7, 8].

Long-term non-progressing HIV-infected adults tend to maintain high CD4 counts in the setting of low viral loads, so-called ‘elite’ controllers having undetectable viraemia [9]. Protective HLA class I molecules such as HLA-B*27, HLA-B*57, HLA-B*58:01 and HLA-B*81:01 are highly enriched amongst adult elite controllers [9–11]. The mechanisms proposed for HLA-associated control of HIV include the ability of these ‘protective’ HLA molecules to present Gag-specific epitopes for recognition by CD8+ T-cells to facilitate rapid killing of virus-infected target cells [12, 13]. In these cases, the location of the epitopes, often in highly conserved regions of the capsid protein, may compromise the ability of the virus to select escape mutants that do not incur substantial costs to viral replicative capacity as a consequence [14]. In contrast, ‘protective’ HLA molecules are not expressed at high frequency in long-term non-progressing children [15]. The reasons for the lack of impact of ‘protective’ HLA in children may relate to immune ontogeny [1, 7] and the inability of young children to generate especially strong Th1 type CD4 T-cell responses. Although HIV-specific CD8+ T-cell responses are detectable from birth in utero infected children [16, 17], these responses usually do not have a significant impact on viral replication, at least for the first years of life [15].

In contrast to HLA type, the viral replicative capacity of transmitted virus has a significant impact on disease progression in both paediatric and adult infection [15, 18, 19]. We here studied the role of HLA and viral replicative capacity in a family including three HIV-infected individuals, all expressing HLA-B*81:01, an HLA allele that is highly protective in adult infection [9–11]. Two of these family members were slow progressor children, the third was the mother of one child and grandmother of the other. In this case, it appears that the latter child was infected by breast milk transmission from the grandmother.

## Methods

### Study subjects

The slow progressor cohort of HIV-infected children has been previously described [15]. Informed consent was provided for participation of the subjects in the study. Ethics approval was given by the University of the Free State Ethics Committee, Bloemfontein, the Biomedical research Ethics Committee, University of KwaZulu-Natal, Durban, and the Oxford Research Ethics Committee. Viral load in chronic infection was measured using the Roche Amplicor version 1.5 assay; CD4^+^ T cell counts were measured by flow cytometry.

Four-digit HLA typing of the Class I locus was performed from genomic DNA as previously described [20] by sequence-based typing at the ASHI^*^ accredited HLA typing laboratory, University of Oklahoma Health Sciences Centre, USA. Exons 2 and 3 of HLA Class I were amplified by locus-specific PCR and then sequenced. Resolution of ambiguities was undertaken according to the ASHI committee recommendations.

Additional viral sequence analyses were performed on a previously described, multi-center cohorts: 1470 African clade C Gag sequences from cohorts based in Durban [21], Bloemfontein [22] and Kimberley [23] South Africa, Zambia and the Thames Valley area of the United Kingdom [23].

### IFN-γ ELISPOT assays

IFN-γ enzyme-linked immunospot (Elispot) assays were performed as previously described [24, 25], using optimally defined epitopes and 18mer overlapping peptides (OLP) with input cells/well ranging from 30,000 to 100,000. The number of specific spot-forming cells (SFC) was calculated by subtracting the mean number of spots in the negative control wells from the number of spots counted in each well. The magnitude of epitope-specific responses was calculated as SFC per million cells.

### Site-directed mutagenesis of NL4-3, SK-254, and SK-254(M)

T186S, L188F, L188F/T190I mutations of HIV-1 Gag sequence were introduced into the HIV-1 subtype B NL4-3 plasmid (pNL4-3) and a patient-derived subtype C HIV-1 Gag-protease sequence (SK-254, GenBank accession number HM593258) respectively by using QuikChange Lightning site-directed mutagenesis kit (Agilent technologies) along with custom-designed mutagenesis forward and reversed primers. The forward primers are shown as follows (mutated codons shown in bold): 5′-CC CCA CAA GAT TTA AAT **AGC** ATG CTA AAC ACA GTG GG-3′ (NL4-3 Gag T186S); 5′-CA CAA GAT TTA AAT ACC ATG **TTC** AAC ACA GTG GGG GGA CAT CA-3′ (NL4-3 Gag L188F); 5′-CCA CAA GAT TTA AAT ACC ATG **TTC** AAC **ATA** GTG GGG GGA CAT CAA GCA G-3′ (NL4-3 Gag L188F/T190I); 5′-CC CCA CAA GAT TTA AAC **AGC** ATG CTA AAT ACA GTG GG-3′ (SK-254 Gag T186S); 5′-CA CAA GAT TTA AAC ACC ATG **TTC** AAT ACA GTG GGG GGA CAT CA-3′ (SK-254 Gag L188F); 5′-CCA CAA GAT TTA AAC ACC ATG **TTC** AAT **ATA** GTG GGG GGA CAT CAA GCA G-3′ (SK-254 Gag L188F/T190I). In addition, considering the six SK-254 variants in p24 Gag might affect viral fitness, we used QuikChange Lightning Multi site-directed mutagenesis kit (Agilent technologies) to retro-mutate these variants to Concensus C. The customised former primers are (mutated codons shown in bold): 5′-G CAA ATG GTA CAC CAA GCC **ATA** TCA CCT AGA ACT TTG AAT G-3′ (SK-254 Gag T147I); 5′-AA ATA GCA TGG ATG ACT **AGT** A AC CCA CCT **ATC** CCA GTG GGA G-3′ (SK-254 Gag G252S/V256I); 5′-ACA CAA GAT GTA AAA AAT TGG ATG ACA **GAT** ACC TTG TTG GTC CAA-3′ (SK-254 Gag E319D); 5′-C ATT TTA AGG GCA TTA GGA CCA **GGA** GCT **ACG** TTA GAA GAA ATG ATG ACA GCA TG-3′ (SK-254 Gag A340G/S342T) Hence, the p24 Gag in this modified SK-254 sequence (SK-254(M)) is the same as Consensus C. The reverse mutagenesis primers comprised the reverse complement of the forward primers shown. The other mutations Q182S, T190A, T190I, T186S/Q182S, T186S/T190A, and T186S/T190I were kindly engineered and provided by Dr. Jaclyn K. Wright (University of KwaZulu-Natal, Durban, South Africa). All the mutations were confirmed by sequencing.

### Virus production and replication kinetics

All the plasmids were maxipreped according to manufacturer’s instruction (HiSpeed^®^ plasmid Maxi Kit, Qiagen, Hilden, Germany) beforehand. To generate the mutant viruses, the mutated NL4-3 and SK-254, SK-254(M) Gag-Pro amplified PCR cleaned up products along with the BstE II (New England Biolabs, Ipswich, MA) linearized pNL4-3Δgag-protease were transfected into GFP reporter GXR cells via electroporation in a BioRad GenePulsar II using 0.4 cm cuvettes at 300 V, 500 µF, and infinite resistance as previously described [14]. Virus propagation was then monitored by flow cytometry to detect GFP-expressing infected cells in approximately 2 weeks in culture with GXR cells. Virus cultures supernatants were harvested mostly when 30 % of cells were GFP-positive. Viruses were aliquoted and stored at −80 °C until use. All the mutations were confirmed again by extracting viral RNA from the harvest supernatant and sequencing. The nucleotide identity was 99.92 %.

Along with WT as positive controls and two negative controls without viruses, NL4-3 and SK-254 mutant viruses were incubated with GXR cells in a 24-well plate for determination of viral titres, as previously described [26, 27]. The percentages of GXR-positive cells were measured by flow cytometry after 48 h. A low MOI (0.03 %) was set as the lowest threshold for determining the amount of virus required for inoculation. The GFP+ expression was measured from day 2 to 7(or 8) before it reached the saturated 30–40 %. The viral replication capacity was defined by the natural log calculation of the mean slope of exponential growth in Excel. This was further calibrated to the normalized value relative to the WT NL4-3, SK-254, and SK-254(M) respectively. All the assays were done at least in triplicate.

### Amplification and sequencing of proviral DNA

Genomic DNA was extracted during the separation of PBMCs and the Gag sequences were then amplified by nested PCR as previously described [28, 29]. The primers of PCR reactions are: 5′-CTCTAGCAGTGGCGCCCGAA-3′ and 5′-TCCTTTCCACATTTCCAACAGCC-3′ for the first round; 5′-CAATTTCTGGCTATGTGCCC-3′ and 5′-ACTCGGCTTGCTGAAGTGC-3′ for the second. After PCR products purification (QIAquick PCR Purification Kit, Qiagen), BigDye Terminator v3.0 Ready Reaction mix (Applied Biosystems) was applied for all the sequencing, with three pairs of primers: 5′-TCT CTC GAC GCA GGA CTC-3′, 5′-TTT CCA CAT TTC CAA CAG CC-3′, 5′-CTG CAC TAT AGG ATA ATT TTG AC-3′, 5′-GAC ACC AAG GAA GCC TTA G-3′, 5′-CTC CCA CTG GAA CAG GTG-3′ and 5′-GGA ACA AAT AGC ATG GAT GAC-3′). The obtained sequences were run on the ABI 3700 DNA analyzer. As previously described [23], Sequences were analyzed by using Sequencher v5.0.1 (Gene Codes Corporation). HXB sequence was used as a reference for all residue numbers.

### Phylogenetic analysis

A maximum likelihood phylogenetic tree using the general time reversible model of nucleotide substitution was constructed with 1000 bootstrap replicates using Mega 7.0.14 software and viewed using FigTree v1.4.0 software. Clade consensus sequence was generated using the Gag-Protease sequence and the Simple Consensus Maker tool available from the Los Alamos HIV database (http://www.hiv.lanl.gov/). Forty-three Gag-Protease C clade reference sequences from Durban, South Africa, from the same locality as the study subjects GM, GD and D2, were included as reference sequences.

### Structural modelling

The p24-Gag protein (from PDB: 1GWP) was used to model known mutations in the virus. Sequences were adjusted with COOT [30] and graphical representations were prepared with PYMOL (The PyMol Molecular Graphics System, Schrodinger, LLC).

## Results

### Identification of a pediatric slow progressor infected via grandmother-to-child transmission

**Fig. 1:**
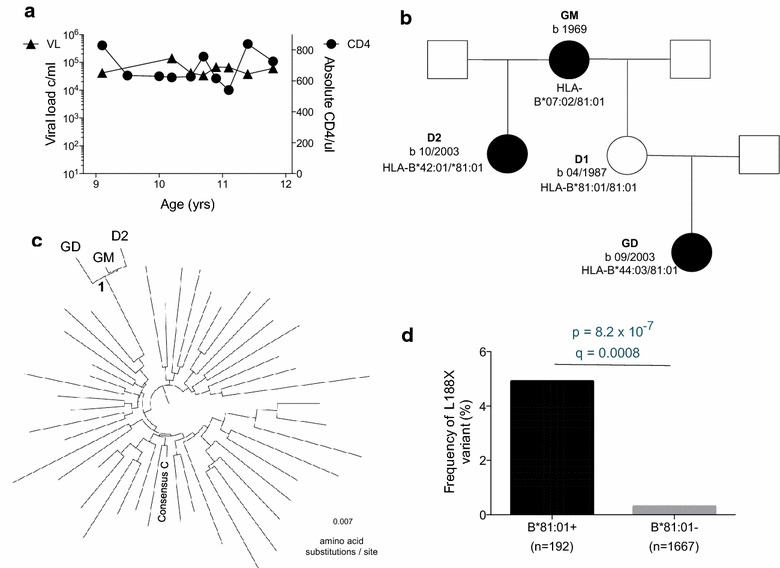
Grandmother-to-child transmission in a non-progressing child. **a** Viral load and absolute CD4 count in non-progressing, ART-naive child GD. **b** Family tree including HIV-infected family members GM (grand-mother), GD (grand-daughter), D2 (second daughter of GM) and uninfected mother of GD, D1. **c** Phylogenetic tree including GD, GM and D2, and 43 HIV-infected adults from the same locality in Durban, South Africa. **d** Frequency of the Gag variant L188F present in all three family members in chronically infected adults with C clade infection (data from Ref. [32])

We have previously defined ‘paediatric slow progressors’ as ART-naïve, HIV-infected children who have not progressed to meet clinical or CD4 count criteria for ART initiation [15]. The CD4 count criteria for ART initiation in children aged >5 years in South Africa until 2013 was an absolute CD4 count of ≤350 cells/ul [15]. Within a study cohort of paediatric slow progressors in Durban, South Africa, a female (‘GD’) was enrolled at age 9.1 years, with an absolute CD4 count of 830 cells/ul and viral load of 42,000 copies/ml (Fig. 1a). The mother (‘D1’) of GD, however, tested HIV-uninfected. It emerged that the HIV-infected grandmother (‘GM’) of GD had a second daughter (‘D2’) who was born 5 weeks after GD (Fig. 1b), and that GM had breast-fed both her daughter (D2) and her grand-daughter (GD). Phylogenetic analysis (Fig. 1c) of HIV Gag sequences obtained from GM, D2 and GD were consistent with mother-to-child transmission of HIV from GM to D2, and with grandmother-to-child transmission of HIV from GM to GD.

### Rare mutation Gag-L188F within HLA-B*81:01-TL9 epitope shared by transmission trio

Both GD and D2 were pediatric slow progressors, as defined by the criteria of reaching 5 years of age, ART-naive, with an absolute CD4 count of >350 ul (Additional file 1: Table S1). In case of D2, ART was initiated at age 6.7 years despite a relatively high CD4 count of 625 cells/mm^3^, but because of TB infection. Factors that might contribute to slow disease progression in related family members would include viral factors and shared host genetic factors. We first sought to test the hypothesis that slow disease progression in GD and D2 might be related to common infection by transmission from GM of a virus with low replicative capacity, since viral replicative capacity has been shown to play an important role in disease progression in both paediatric and adult HIV infection [15, 18], We focused on the Gag region, since previous studies have demonstrated a strong correlation between Gag mutants, viral replicative capacity and disease outcome in both adults and pediatric infection [15, 18, 31]. The virus in all family members GM, D2 and GD expressed the mutation Gag-L188F (Table 1). This mutant arises at the carboxy-terminal anchor residue of the HLA-B*81:01-TL9 epitope (TPQDLNTML, Gag 180–188). This variant is rare, being observed in <0.2 % of published sequences (http://www.hiv.lanl.gov/) but is strongly associated with expression of HLA-B*81:01 from previous large cohort studies of C clade infection [32] (Fig. 1d, p = 6.3 × 10^−7^, q = 0.0008). These data are consistent either with L188F being an escape mutant driven by the HLA-B*81:01-TL9 response in GM and transmitted to both GD and D2; or being selected independently in each of the three family members, all of whom express HLA-B*81:01 (Additional file 1: Table S1).

**Table 1:**
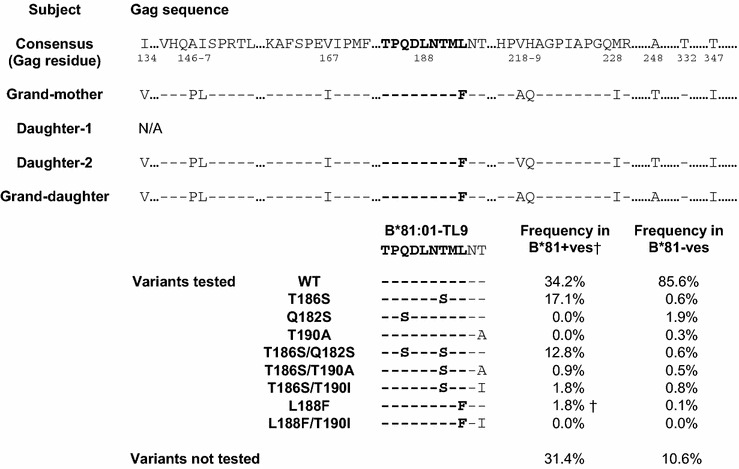
Differences in autologous Gag sequences between grandmother, grand-daughter and daughter-2, TL9 variants tested and their frequency in HIV-infected adults

^†^The frequency is of the variant shown only; for example other variants in combination with L188F such as Q182S/L188F are found in the study cohort but these variants were not tested

### Impact of L188F on viral replicative capacity

Previous studies [27, 33] have demonstrated that the most common escape variants within the HLA-B*81:01-TL9 epitope, T186S and Q182S/T186S (Table 1), can have a substantial impact on viral replicative capacity (VRC). To investigate the possibility that this rare L188F mutant might similarly affect VRC, thereby contributing to slow progression observed in subjects GD and D2, this variant was generated by site-directed mutagenesis and the replicative capacity compared with the more common and well-described HLA-B*81:01-associated TL9 escape mutants, T186S, the combination of T186S/Q182S, and the putative compensatory mutants T190A and T190I (33; Table 1). These comparisons were undertaken using 3 different viral backbones: the laboratory-adapted B clade virus NL4-3; a chimeric virus comprising NL4-3 with Gag-Pro inserted from a C clade infected South African subject SK-254, whose viral sequence is similar to the C clade consensus [33]; and a chimeric virus, referred to here as SK-254(M), comprising NL4-3 with the same Gag-Pro inserted from the C clade infected South African subject SK-254, but with the necessary retro-mutations made to enable the viral amino acid sequence to be identical with the C clade consensus (Additional file 2: Table S2).

**Fig. 2:**
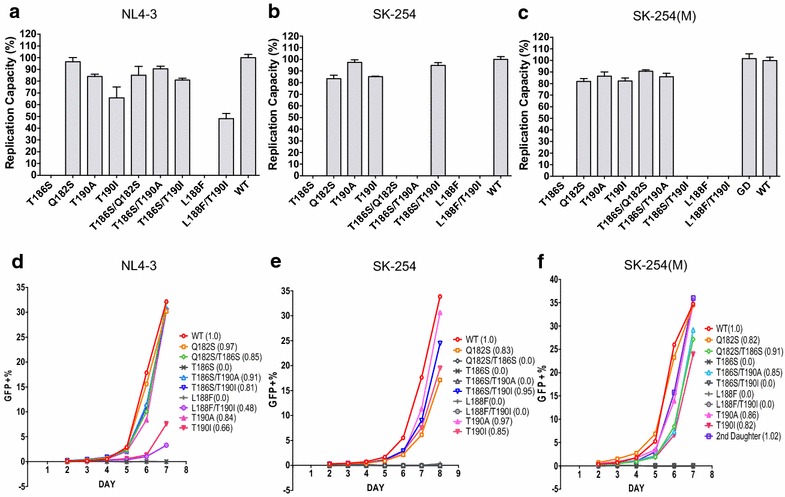
Impact of variants within the HLA-B*81:01-TL9 epitope on viral replicative capacity (VRC). **a**–**c** Percentage GXR cells expressing GFP following infection with an MOI of 0.03 using virus variants shown. **d**–**f** Impact of viral variants studied on replication capacity compared to NL4-3 in three distinct viral backbones

The VRC determined for each mutant virus is shown relative to NL4-3, SK-254, and SK-254(M) respectively (Fig. 2). Irrespective of the backbone, any viruses encoding L188F or T186S were incapable of replicating at a sufficient level in vitro to measure VRC. Although GFP+ expression in target cells remained negative up to >100 days in culture, viral RNA was successfully extracted from the supernatant and the respective L188F and T186S mutations, confirmed by sequencing. This would indicate that the viruses were generated and replicating, although insufficiently to assay VRC. The second observation here is that the viral backbone has a considerable influence on the impact that individual variants may make on VRC. For example, T190I shows a modest compensatory effect in combination with L188F in NL4-3, but not in SK-254 or SK-254(M). In relation to T186S, T190A and T190I are compensatory in the setting of NL4-3, but only T190I in the context of SK254 and only T190A in the context of SK254(M). Of note, however, the entire Gag-Pro sequence taken from GD to form a chimeric virus with NL4-3 replicated as well as SK-254(M) WT, despite containing the L188F variant. This indicates that one or more of the additional variants within the Gag-Pro sequence of the GD were capable of compensating for L188F. We were not able to generate chimeric viruses from the GM or D2 samples available but these differed in sequence by only two and one residue from that of GD, respectively (Table 1).

### Structural analysis indicates likely mechanism of L188F and T186S impact on VRC

**Fig. 3:**
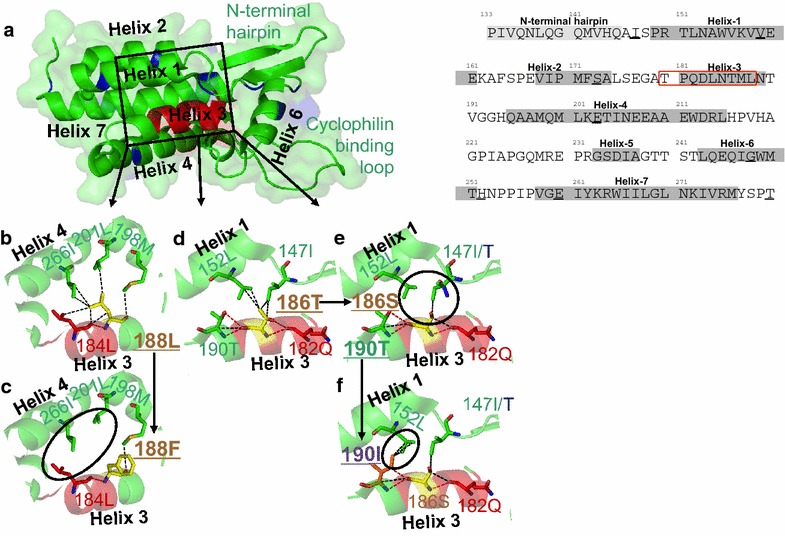
Structural modelling of the impact of escape mutations around the p24-Gag B81:01-TL9 epitope. **a** Overall structure of p24-Gag protein residues 133–283 demonstrating the position of the B*81:01-TL9 epitope (*red cartoon*) and the polymorphisms between the NL4-3 and SK-254 viruses (*blue cartoon*). The environment that is likely to be affected by mutations around the B*81:01-TL9 epitope, in helix 3, is highlighted in the *black box*. **b** Binding network (*green* and *red sticks*) around residue 188L (*yellow sticks*) in the NL4-3 virus, likely to be important for maintaining the protein fold between helix 3 and 4. **c** Modelling of the rare L188F mutation, shown to reduce viral replication capacity. This mutation could abrogate interactions between residue 188 with 184L, 198 M, 201L and 266I, possibly destabilizing the helix 3–helix 4 interface. **d** Binding network (*green* and *red sticks*) around residue 186T (*yellow sticks*) in the NL4-3 virus, likely to be important for maintaining the protein fold between helix 1 and 3. **e** Modelling of the T186S mutation (*black arrow*), shown to be detrimental to viral health. This mutation abrogates interactions between residue 186 with 152L and 147I/T (*black circle*), possibly destabilizing the helix 1–helix 3 interface. **f** Structural modeling of the T190I mutation (*black arrow*) that can rescue the T186S mutation, restoring viral replication capacity. Modelling suggests that new interactions can form between residue 190I and 152L (*black circle*), potentially restoring helix 1–helix 3 interface stability

In order to better understand why the HLA-B*81:01-driven escape mutants L188F and T186S have such a marked impact on VRC, we analyzed these mutants using previously solved crystal structures of the capsid protein [34–37]. The capsid protein comprises an amino-terminal domain (NTD: Gag 133–283) linked to a carboxy-terminal domain (CTD: Gag 284–363); the main features of the NTD are an N-terminal beta-hairpin loop, 7 α-helices, and the cyclophilin binding loop linking helices-4 and-5 (Fig. 3a). The TL9 epitope (Gag 180–188) is located almost exactly within helix-3 (Gag residues 181–189).

To consider the impact of the L188F escape mutant, the contacts between 188-Leu and neighboring residues 184L, 198M, 201L and 266I were identified, these forming 7 van der Waals interactions, in addition to the hydrogen bond between the backbone amine group at residue 188 and the backbone carbonyl group at residue 184 (Fig. 3b). Modeling of the L188F substitution would indicate that all but one of these 7 van der Waals interactions are disrupted (Fig. 3c), thereby likely substantially destabilizing the interface between helices-3 and -4. Similarly, modeling would suggest that the consequence of the T186S escape mutant would be to abrogate 3 of 4 van der Waals interactions between 186-Thr on helix-3 and 152-Leu and 147-Ile on helix-1 (Fig. 3d). The destabilizing impact of this would, however, be largely mitigated by the compensatory T190I mutation, in providing 3 new van der Waals interactions between 190-Ile and 152-Leu (Fig. 3e). However, the variable ability of variants at 190-Thr to compensate successfully for the fitness cost of the T186S escape mutant according to viral backbone (NL4-3, SK-254 or SK-254(M)) is not explained by these models, with the majority of the differences between these viruses located within the carboxy-terminal domain (Additional file 2: Table S2).

## Discussion

These studies describe two HIV-infected, genetically related children, born within 5 weeks of one another, both of whom progressed slowly to disease. One child was infected via mother-to-child transmission and the other, more unusually, was infected by the same donor, but via grandmother-to-child transmission. This scenario raised the question of whether slow progression in the two genetically related children could be related to the virus transmitted, or to shared host factors. The donor, ‘GM’, the grand-daughter, ‘GD’, and the HIV-infected daughter, ‘D2’, all expressed HLA-B*81:01, which is normally associated with protection against disease progression in adult infection [10, 11]. Autologous virus in each of the three family members also shared the same, rare, L188F mutation, which is strongly associated with HLA-B*81:01, and lies at the carboxy-terminus of the immunodominant HLA-B*81:01-restricted Gag epitope TL9 (TPQDLNTML, Gag 180–188). In common with the more frequent HLA-B*81:01-TL9 escape mutation T186S, the L188F variant almost completely prevented viral replication when introduced into heterologous HIV strains. However, the replicative capacity of virus encoding the entire grand-daughter (GD) Gag sequence did no differ from wildtype virus. It remains entirely possible (as discussed further below) that either the uncompensated L188F variant was transmitted to both daughters, or that wildtype virus was transmitted and L188F plus the near-identical compensatory mutants were selected independently post-transmission in GM, GD and D2. However, on the basis that low fitness viruses are less likely to be transmitted [32], the most parsimonious interpretation of these data may be that the fully compensated L188F virus was transmitted in both cases and that slow progression in the two children was not related to viral factors but to undefined host-specific factors.

Previous studies of the mutations selected within the HLA-B*81-TL9 epitope have similarly shown that the most frequent escape variant, T186S, can virtually abrogate viral replication [33]. Similar to the analyses reported here, the impact of T186S on viral replicative capacity varied considerably according to the nature of the viral backbone, as did the ability of variants such as T190I to rescue the virus from the fitness cost resulting from T186S [33]. The studies described here are highly consistent with these findings, in demonstrating, first, the dramatic negative impact of L188F, as well as T186S, on viral replicative capacity; and, second, the striking influence of viral sequence context. For example, T190I and not T190A was able to compensate for T186S in the SK-254 virus but the reverse was the case in the SK-254(M) virus. The p24 Gag residues that might contribute to the substantial influence of sequence context on the impact of the TL9 escape mutants on viral replicative capacity observed here include several that have been previously implicated as compensatory mutants [26, 28, 38, 39], such those within the cyclophilin A binding loop (V218A, H219Q and M228T), and the Gag tropism-determining loops (Gag residues 137–147; and Gag 248–254) [40].

The lack of samples at the time of transmission means that the sequences of the viruses that were transmitted are unknown. It is possible therefore that the same L188F variant within the B*81:01-TL9 epitope was selected independently from the wildtype TL9 sequence in all three family members subsequent to transmission, since all three individuals expressed HLA-B*81:01. If this were the case, it is likely that the virus transmitted by GD without L188F would have had normal viral replicative capacity, at least on the basis of the otherwise unremarkable *gag* sequence in these three family members. In the more likely scenario of the virus encoding L188F being transmitted both to GD and D2, the fact that this virus was fully compensated in GD suggests that low viral replicative capacity is unlikely to have contributed to slow progression in the two children.

These observations would suggest therefore that undefined host factors are more likely to have contributed to slow progression in the two genetically related children. Although in this case both children expressed HLA-B*81:01 that is associated with slow progression in adult infection, large cohort studies indicate that ‘protective’ HLA alleles, such as HLA-B*57, HLA-B*58:01 and HLA-B*81:01, do not significantly influence paediatric disease progression [15]. Furthermore, in a small series of HLA-B*27-positive HIV-infected children, any benefit to the child of expressing HLA-B*27 was negated in instances where the HLA-B*27-mother transmitted an escape mutant in the critical HLA-B*27 Gag epitope [41]. In the current study, no HLA-B*81:01-TL9 response was detectable in the child GD (Additional file 3: Figure S1), suggesting that this response is not critical to non-progression. Furthermore, in keeping with other non-progressor children [8, 42], normal-for-age CD4 counts were maintained in GD through childhood despite relatively high viral loads (between 38,000 and 140,000 between 9 and 12 years of age). HLA-mediated control of viremia that is characteristic of adult non-progression [12] is not a typical feature of non-progressive paediatric infection. In common with the natural hosts of SIV in whom normal CD4 counts and low systemic immune activation are also maintained despite persistent high level viremia, the mechanisms underlying non-progression in paediatric infection differ very substantially from those operating among adult elite controllers [9] and remain incompletely defined.

One additional notable feature in this study is the occurrence of paediatric HIV infection in the absence of mother-to-child transmission. The particular scenario of grandmother-to-child transmission has not, to our knowledge, been described previously, but transmission via surrogate breast-feeding is a well-recognized cause of non-vertical, non-sexual HIV infection in children [43, 44]. The prevalence of paediatric transmission via surrogate breast-feeding is unknown but, from data accumulated within large studies of >250 infected children, this would likely represent approximately 1–2 % of paediatric infections [45–47]. The relevance of grandmother-to-child to non-progression is open to speculation, but certainly timing of transmission affects outcome in paediatric infection, in utero infected children progressing faster than those infected *intra*-*partum*, and children infected *post*-*partum* via breast milk progressing the slowest [48].

## Conclusion

These data are consistent with previous studies indicating that, compared to adult elite controllers, distinct mechanisms underlie slow HIV paediatric disease progression. The principal host factors responsible for disease non-progression in children appear not to include HLA class I and remain yet to be defined. Further evaluation of this group of paediatric slow progressors is therefore warranted.

## Additional files

10.1186/s12977-016-0300-y HLA types and clinical data available for Grand-mother, Grand-daughter, Daughter-1 and Daughter-2.
10.1186/s12977-016-0300-y p17 + p24 Gag sequences for NL4-3, SK-254, SK-254(M) and Consensus C clade.
10.1186/s12977-016-0300-y IFN-gamma Elispot responses of PBMC in Grand-mother and Grand-daughter to a panel of 410 overlapping peptides spanning the C clade proteome. Responses to the overlapping peptides as shown. The immunodominant HLA-B*81:01 epitope TL9 is contained within overlapping peptide (OLP)-25, there was no response to this in either GM or GD.

## Abbreviations

ART: antiretroviral therapy
CTD: carboxy-terminal domain
D1: daughter-1
D2: daughter-2
GM: grand-mother
GD: grand-daughter
NTD: amino-terminal domain
VRC: viral replicative capacity
