# Supplementary material for: Paediatric non-progression following grandmother-to-child HIV transmission
Source: Retrovirology. 2016 Sep 8;13(1):65. doi: 10.1186/s12977-016-0300-y (PMC5016918; doi:10.1186/s12977-016-0300-y)
Supplement: Supplementary file 1 — 10.1186/s12977-016-0300-y HLA types and clinical data available for Grand-mother, Grand-daughter, Daughter-1 and Daughter-2. [file 12977_2016_300_MOESM1_ESM.docx]

**Additional file 1**

| **Subject** | **ID** | **Sex** | **dob** | **HIV status** | **ART initiation** | **Age* (yrs)** | **Pre-ART CD4** | **Pre-ART CD4%** | **Pre-ART Viral Load** | **HLA-B type** |
| --- | --- | --- | --- | --- | --- | --- | --- | --- | --- | --- |
| **Grand-mother** | **GM** | f | 12/68 | pos | 05/04 | 35.4 | 329† | 32%† | <50† | 07:02/81:01 |
| **Daughter-1** | **D-1** | f | 04/87 | neg | n/a | n/a | nd | nd | nd | 81:01/81:01 |
| **Daughter-2** | **D-2** | f | 10/03 | pos | 06/10 | 6.7 | 625 | 27% | n/a | 42:01/81:01 |
| **Grand-daughter** | **GD** | f | 09/03 | pos | ART-naive | 9.1 | 830 | 33% | 42,000 | 44:03/81:01 |

* Age at ART initiation, or in grand-daughter GD age at enrollment

† pre-ART CD4 counts and viral load not available for Grand-mother GM; data show are CD4 counts and viral loads after 23m on ART in 04/2006 when GM was aged 37.3yrs.

**Table S1. HLA types and clinical data available for Grand-mother, Grand-daughter, Daughter-1 and Daughter-2.**
