# Supplementary material for: Paediatric non-progression following grandmother-to-child HIV transmission
Source: Retrovirology. 2016 Sep 8;13(1):65. doi: 10.1186/s12977-016-0300-y (PMC5016918; doi:10.1186/s12977-016-0300-y)
Supplement: Supplementary file 2 — 10.1186/s12977-016-0300-y p17 + p24 Gag sequences for NL4-3, SK-254, SK-254(M) and Consensus C clade. [file 12977_2016_300_MOESM2_ESM.docx]

**Additional file 2**

|  | |  |
| --- | --- | --- |
| **Plasmid** | **HXB2 Gag position** |  |
|  | **147 159** | |
| **NL4-3** | **P I V Q N L Q G Q M V H Q A I S P R T L N A W V K V V E E K A F S P E V I P M F** | |
| **SK-254** | **- - - - - - - - - - - - - - T - - - - - - - - - - - I - - - - - - - - - - - - -** | |
| **SK-254(M)** | **- - - - - - - - - - - - - - - - - - - - - - - - - - I - - - - - - - - - - - - -** | |
| **Consensus C** | **- - - - - - - - - - - - - - - - - - - - - - - - - - I - - - - - - - - - - - - -** | |
|  | **173 203** | |
| **NL4-3** | **S A L S E G A T P Q D L N T M L N T V G G H Q A A M Q M L K E T I N E E A A E W** | |
| **SK-254** | **T - - - - - - - - - - - - - - - - - - - - - - - - - - - - - D - - - - - - - - -** | |
| **SK-254(M)** | **T - - - - - - - - - - - - - - - - - - - - - - - - - - - - - D - - - - - - - - -** | |
| **Consensus C** | **T - - - - - - - - - - - - - - - - - - - - - - - - - - - - - D - - - - - - - - -** | |
|  | **248 252** | |
| **NL4-3** | **D R L H P V H A G P I A P G Q M R E P R G S D I A G T T S T L Q E Q I G W M T H** | |
| **SK-254** | **- - - - - - - - - - - - - - - - - - - - - - - - - - - - - - - - - - - A - - - G** | |
| **SK-254(M)** | **- - - - - - - - - - - - - - - - - - - - - - - - - - - - - - - - - - - A - - - S** | |
| **Consensus C** | **- - - - - - - - - - - - - - - - - - - - - - - - - - - - - - - - - - - A - - - S** | |
|  | **256 260 280 286** | |
| **NL4-3** | **N P P I P V G E I Y K R W I I L G L N K I V R M Y S P T S I L D I R Q G P K E P** | |
| **SK-254** | **- - - V - - - D - - - - - - - - - - - - - - - - - - - V - - - - - K - - - - - -** | |
| **SK-254(M)** | **- - - - - - - D - - - - - - - - - - - - - - - - - - - V - - - - - K - - - - - -** | |
| **Consensus C** | **- - - - - - - D - - - - - - - - - - - - - - - - - - - V - - - - - K - - - - - -** | |
|  | **301 310 312 319** | |
| **NL4-3** | **F R D Y V D R F Y K T L R A E Q A S Q E V K N W M T E T L L V Q N A N P D C K T** | |
| **SK-254** | **- - - - - - - - F - - - - - - - - T - D - - - - - - - - - - - - - - - - - - - -** | |
| **SK-254(M)** | **- - - - - - - - F - - - - - - - - T - D - - - - - - D - - - - - - - - - - - - -** | |
| **Consensus C** | **- - - - - - - - F - - - - - - - - T - D - - - - - - D - - - - - - - - - - - - -** | |
|  | **335 340 342 357** | |
| **NL4-3** | **I L K A L G P G A T L E E M M T A C Q G V G G P G H K A R V L** | |
| **SK-254** | **- - R - - - - A - S - - - - - - - - - - - - - - S - - - - - -** | |
| **SK-254(M)** | **- - R - - - - - - - - - - - - - - - - - - - - - S - - - - - -** | |
| **Consensus C** | **- - R - - - - - - - - - - - - - - - - - - - - - S - - - - - -** | |

**Table S2. P24 Gag sequences for NL4-3, SK-254, SK-254(M) and Consensus C clade.**
