# Supplementary material for: Paediatric non-progression following grandmother-to-child HIV transmission
Source: Retrovirology. 2016 Sep 8;13(1):65. doi: 10.1186/s12977-016-0300-y (PMC5016918; doi:10.1186/s12977-016-0300-y)
Supplement: Supplementary file 3 — 10.1186/s12977-016-0300-y IFN-gamma Elispot responses of PBMC in Grand-mother and Grand-daughter to a panel of 410 overlapping peptides spanning the C clade proteome. Responses to the overlapping peptides as shown. The immunodominant HLA-B*81:01 epitope TL9 is contained within overlapping peptide (OLP)-25, there was no response to this in either GM or GD. [file 12977_2016_300_MOESM3_ESM.pptx]

## Slide 1
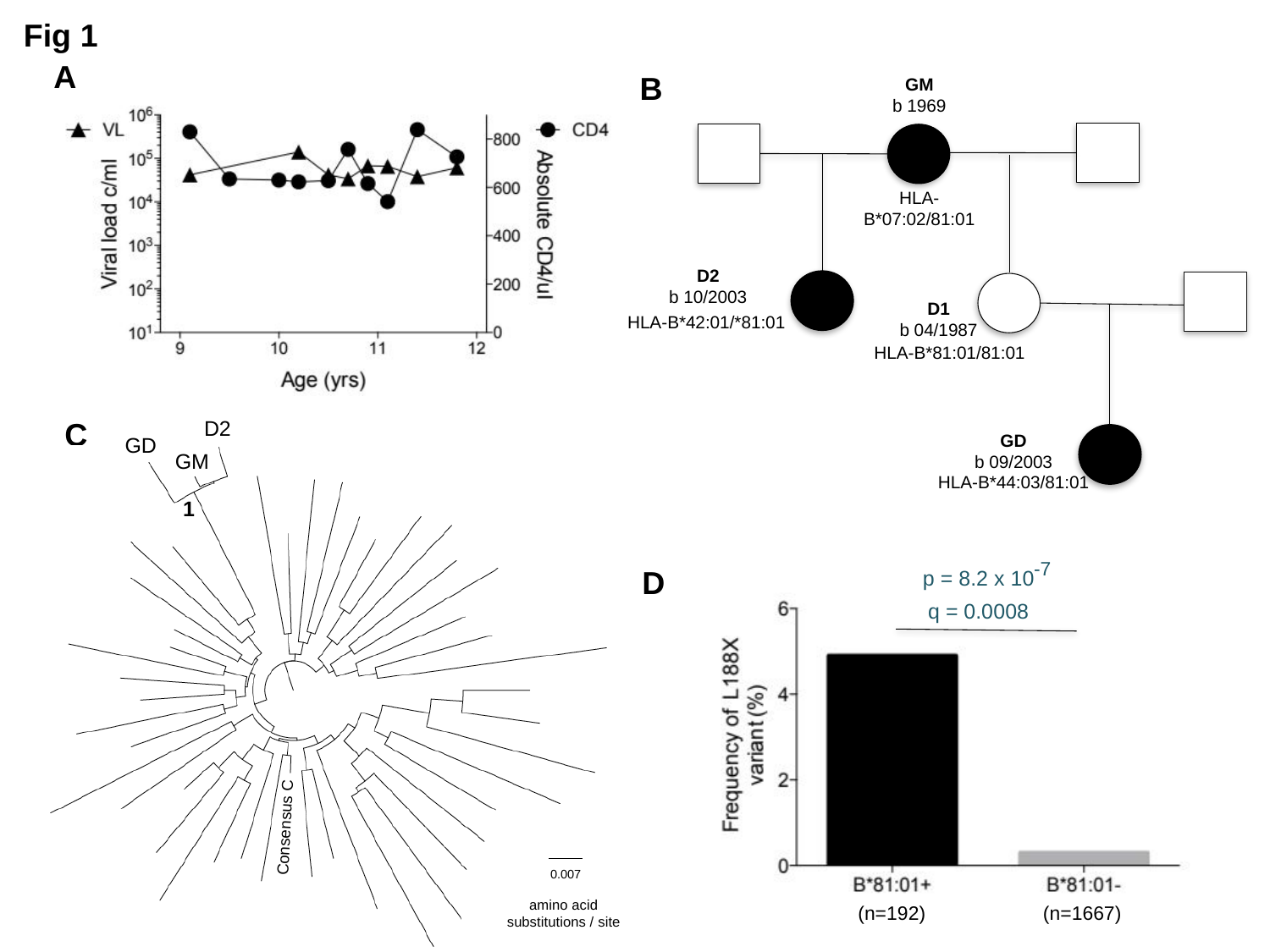

Fig 1
A
B
GM
b 1969
HLA-B*07:02/81:01
D2
b 10/2003
D1
b 04/1987
HLA-B*42:01/*81:01
HLA-B*81:01/81:01
C
D2
GD
GM
1
Consensus C
0.007
amino acid
substitutions / site
GD
b 09/2003
HLA-B*44:03/81:01
-7
p = 8.2 x 10
D
q = 0.0008
(n=192)
(n=1667)

## Slide 2
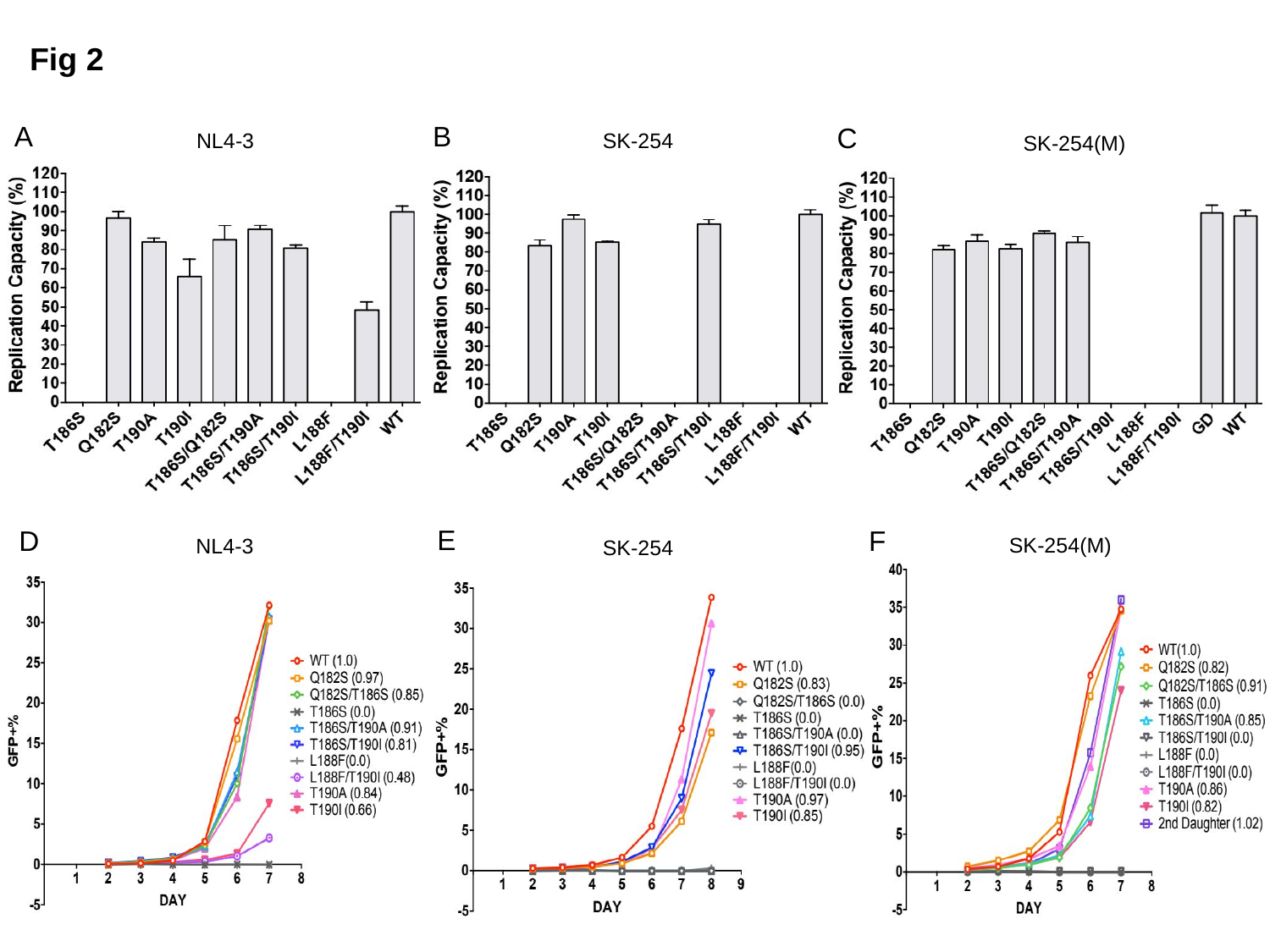

Fig 2
A
B
C
NL4-3
SK-254
SK-254(M)
E
D
F
SK-254(M)
NL4-3
SK-254

## Slide 3
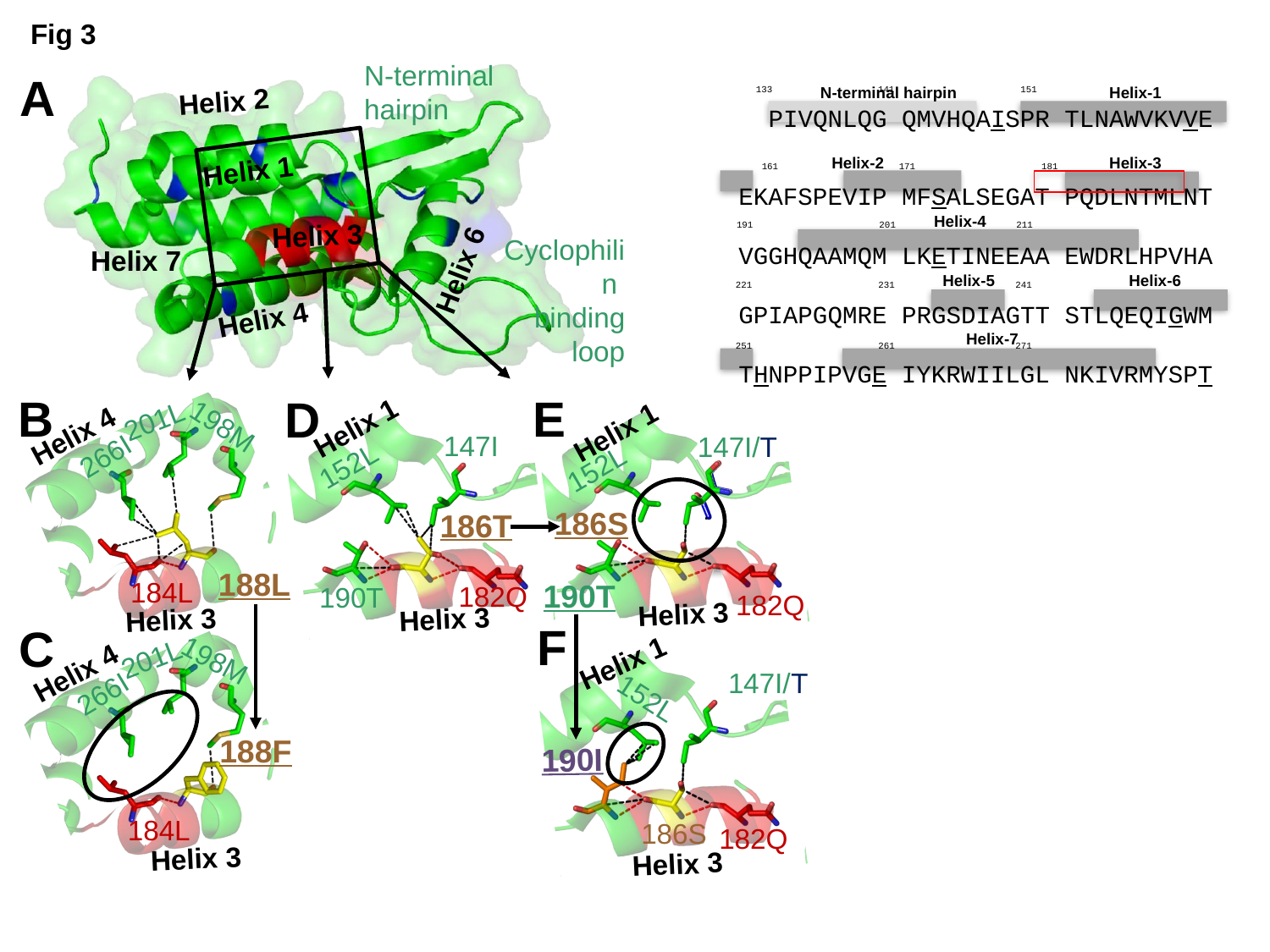

Fig 3
N-terminal
hairpin
A
 133 141 151
 PIVQNLQG QMVHQAISPR TLNAWVKVVE
EKAFSPEVIP MFSALSEGAT PQDLNTMLNT
VGGHQAAMQM LKETINEEAA EWDRLHPVHA
GPIAPGQMRE PRGSDIAGTT STLQEQIGWM
THNPPIPVGE IYKRWIILGL NKIVRMYSPT
N-terminal hairpin
Helix-1
Helix 2
Helix-2
Helix-3
Helix 1
161 171 181
Helix-4
191 201 211
Helix 3
Cyclophilin
binding loop
Helix 7
Helix 6
Helix-6
Helix-5
221 231 241
Helix 4
Helix-7
251 261 271
B
E
D
201L
198M
Helix 1
Helix 1
Helix 4
147I
147I/T
266I
152L
152L
186S
186T
188L
184L
190T
182Q
190T
182Q
Helix 3
Helix 3
Helix 3
F
C
201L
198M
Helix 1
Helix 4
147I/T
266I
152L
188F
190I
184L
186S
182Q
Helix 3
Helix 3
